# Prediction of Failure to Progress after Labor Induction: A Multivariable Model Using Pelvic Ultrasound and Clinical Data

**DOI:** 10.3390/jpm14050502

**Published:** 2024-05-09

**Authors:** Blanca Novillo-Del Álamo, Alicia Martínez-Varea, Elena Satorres-Pérez, Mar Nieto-Tous, Fernando Modrego-Pardo, Carmen Padilla-Prieto, María Victoria García-Florenciano, Silvia Bello-Martínez de Velasco, José Morales-Roselló

**Affiliations:** 1 Department of Obstetrics and Gynecology, La Fe University and Polytechnic Hospital, 46026 Valencia, Spain; novillo_bla@gva.es (B.N.-D.Á.); satorres_ele@gva.es (E.S.-P.); nieto_martou@gva.es (M.N.-T.); modrego_ferpar@gva.es (F.M.-P.); padilla_carpri@gva.es (C.P.-P.); garcia_mariavictoriaflo@gva.es (M.V.G.-F.); bello_sil@gva.es (S.B.-M.d.V.); jose.morales@uv.es (J.M.-R.); 2 Department of Pediatrics, Obstetrics and Gynecology, Faculty of Medicine, University of Valencia, 46010 Valencia, Spain; 3 Department of Medicine, CEU Cardenal Herrera University, 12006 Castellón de la Plana, Spain; 4 Faculty of Health Sciences, Universidad Internacional de Valencia, 46002 Valencia, Spain

**Keywords:** labor induction, vaginal delivery, cesarean section, pregnancy, pelvic ultrasound

## Abstract

**Objective:** Labor induction is one of the leading causes of obstetric admission. This study aimed to create a simple model for predicting failure to progress after labor induction using pelvic ultrasound and clinical data. **Material and Methods:** A group of 387 singleton pregnant women at term with unruptured amniotic membranes admitted for labor induction were included in an observational prospective study. Clinical and ultrasonographic variables were collected at admission prior to the onset of contractions, and labor data were collected after delivery. Multivariable logistic regression analysis was applied to create several models to predict cesarean section due to failure to progress. Afterward, the most accurate and reproducible model was selected according to the lowest Akaike Information Criteria (AIC) with a high area under the curve (AUC). **Results:** Plausible parameters for explaining failure to progress were initially obtained from univariable analysis. With them, several multivariable analyses were evaluated. Those parameters with the highest reproducibility included maternal age (*p* < 0.05), parity (*p* < 0.0001), fetal gender (*p* < 0.05), EFW centile (*p* < 0.01), cervical length (*p* < 0.01), and posterior occiput position (*p* < 0.001), but the angle of descent was disregarded. This model obtained an AIC of 318.3 and an AUC of 0.81 (95% CI 0.76–0.86, *p* < 0.0001) with detection rates of 24% and 37% for FPRs of 5% and 10%. **Conclusions:** A simplified clinical and sonographic model may guide the management of pregnancies undergoing labor induction, favoring individualized patient management.

## 1. Introduction

Labor induction is one of the leading causes of obstetric admission [1]. Ultrasound examination is a valuable tool to establish indications of elective cesarean section (CS) prior to the onset of labor, such as breech presentation, transverse situation, fetal growth restriction, placenta previa, and vasa previa. However, it would also be interesting to anticipate the probability of CS after the onset of uterine contractions [2], as this might be applied to adjust labor management and avoid unnecessary interventions.

CS during labor occurs under two circumstances: failure to progress (FP), the most frequent cause of intrapartum CS; and intrapartum fetal compromise due to intrapartum hypoxia, usually caused by cord compression or placental insufficiency. This work aims to evaluate FP to develop a simple and reproducible model to predict CS prior to labor induction based on clinical and sonographic data.

Progression of labor has been classically evaluated with clinical examination. However, this methodology presents a high inter-operator variability [2]. Several abdominal and transperineal ultrasound parameters have been described to objectively evaluate the fetal head descent: the head perineum distance, the head station (angle of progression), and the head direction; the head rotation—midline angle; and the head flexion—the occiput spine angle and the chin to chest angle [3,4]. Ultrasound is useful to assess the fetal presentation and position, the mother’s cervical length, and the assessment of the maternal pelvic floor dimensions [3].

In this work, we used ultrasound to improve accuracy with regard to the obstetrical aspects that have been classically managed by physical examination: the fetus as the object of delivery, its position in relation to the birth canal, and the appropriate modifications of its soft parts to allow the future descent of the fetus’s head in the pelvis, and, therefore, the probability of a successful vaginal delivery.

## 2. Material and Methods

This was an observational prospective study that included singleton term pregnancies with unruptured amniotic membranes who were admitted to the Labor and Delivery Unit of La Fe University and Polytechnic Hospital for labor induction between March and December 2023. Inclusion criteria were singleton pregnancies with vertex presentation undergoing induction for maternal or fetal reasons, except rupture of membranes. Patients were subdivided according to the induction method as per hospital protocol. In case of favorable Bishop at admission (≥6), direct oxytocic induction (Syntocynon^®®^ Alfasigma, S.p.A. Pomezia (Roma). ITAL) was carried out, while in case of unfavorable Bishop at admission (<6), an initial cervical ripening with PgE2, dinoprostone (Propess^®®^ Ferring GmbH. Kiel. GERMANY), or a mechanical balloon (Cook^®®^ Cook Medical. Limerick. IRELAND) was used up to 12 h. The mechanical balloon was applied in case of previous CS, fetal growth restriction, smallness for gestational age (GA), and maternal asthma or cardiopathy. Finally, if cervical ripening was unsuccessful, it was followed by oxytocic induction according to the hospital protocol [5].

Exclusion criteria discarded patients with labor contractions or ruptured membranes, as well as twin pregnancies, elective CS, stillbirths, and patients with pelvic or other pathologies that contraindicate vaginal delivery.

Despite the study’s observational nature, informed consent was signed by the patients according to the hospital’s Research Ethics Committee approval (CVS F6SCFZZK:TI7B5L3Z:NNHFUYB9).

Four ultrasonographic parameters representing the components of the birth process (the fetus and its position in relation to the pelvis) were collected at admission and before the onset of contractions. The estimated fetal weight (EFW) represented the suitability of the object of delivery. The occiput position represented the suitability of the baby’s position in relation to the pelvis [6,7]. The angle of progression or angle of descent represented the descent of the head in the pelvis. Finally, the cervical length represented the suitability of the soft parts of the birth canal.

EFW was obtained by transabdominal ultrasound, measuring the head circumference, biparietal diameter, abdominal circumference, and femur length according to Hadlock’s equation [8]. The occiput position was established by evaluating either the position of the spine or the fetal eyes. The angle of descent was calculated using transperineal ultrasound, using the angle between the plane passing through the greater diameter of the pubis symphysis and the plane passing through the lower limit of the head. Finally, the cervical length was measured transvaginally with an empty bladder according to the ISUOG guidelines [9].

CS due to FP included three scenarios, in accordance with national and La Fe Hospital guidelines [5]:

1. Failure of induction, defined as the failure to reach the active period of labor (4 cm of dilatation and complete cervical effacement) after twelve hours of oxytocic induction and regular uterine contractions (at least four every ten minutes).
2. Arrest disorder intrapartum, defined as the absence of progress (4 h of arrest) in the cervical dilatation once the active period of labor is reached.
3. Cephalo-pelvic disproportion, defined as the absence of further descent (3 h of arrest in multiparous and 4 h of arrest in primiparous women under epidural analgesia).

Clinical and epidemiological data included maternal age, weight, height, body mass index, number of gestations, parity, number of CS and abortions, last menstrual period, gestational age at admission, interval to 42 weeks (subtraction from 42 weeks of the gestational age at admission), smoking habits, ethnicity, reason for induction, and type of induction (mechanical ripening use of prostaglandins and direct oxytocin induction).

Finally, after birth, data about the length of induction, type of delivery (vaginal, instrumental, CS), APGAR at 5 min, arterial cord pH, birth weight, fetal gender, and baby destination (maternal ward, neonatal ward, and neonatal intensive care unit) were collected.

To evaluate the relationship between the above-mentioned parameters and the study outcomes: regarding CS for FP (including in the analysis all the patients who had a CS due to any of the three criteria of FP described above) and length of induction, a univariable regression analysis was initially performed to select plausible determinants. Afterward, a multivariable regression analysis was carried out to create different models that were evaluated considering the area under the curve (AUC) and the Akaike Information Criteria (AIC). In these models, the AIC and AUC with their 95% confidence interval (CI), *p*-value, and detection rates for false positive rates of 5% and 10% were provided. The best prediction model was selected, not only relying on a high AUC, but also on the lowest AIC. AIC allows a good balance between parsimony and goodness of fit as this study aimed not only to find an accurate model, but also a reproducible one. Statistics and graphs were performed using Graph Pad Prism 9^®®^ and Stat Plus Pro 7^®®^ Free version for Apple Macintosh. Statistical significance was set at *p* < 0.05.

## 3. Results

A total of 387 patients met the inclusion criteria. The clinical and demographic characteristics of the patients are shown in Table 1a, and the outcomes are shown in Table 1b. The median age of the patients was 34 years. Of them, 19.6% underwent a CS for FP, 8% had a CS for abnormal CTG, 51.2% had a spontaneous vaginal delivery, and 21.2% had an instrumental vaginal delivery. Finally, newborns from only 0.8% of the included patients needed to be transferred to an intensive care unit (Table 1b).

Patients with a CS due to FP were significantly younger than those that delivered vaginally (*p* < 0.05), had a lower number of gestations and parity (*p* < 0.0001), and presented a higher induction length (*p* < 0.0001), EFW, EFW centile (*p* < 0.01), birth weight, and birth weight centile (*p* < 0.05). In addition, they had a longer cervical length (*p* < 0.01), a lower angle of descent (*p* < 0.05), and a more frequent posterior occiput position (*p* < 0.0001).

Table 2 shows the univariable analysis, including all plausible parameters for predicting CS for FP. Of them, only maternal age (*p* < 0.05), parity (*p* < 0.0001), EFW centile (*p* < 0.01), cervical length (*p* < 0.01), angle of descent (*p* < 0.05), and posterior occiput position (*p* < 0.01) presented statistical significance. Contrarily, neither fetal gender nor maternal anthropomorphic data nor proximity to 42 weeks were significant. The highest AUC was achieved with maternal parity (AUC = 0.67).

**Figure 1:**
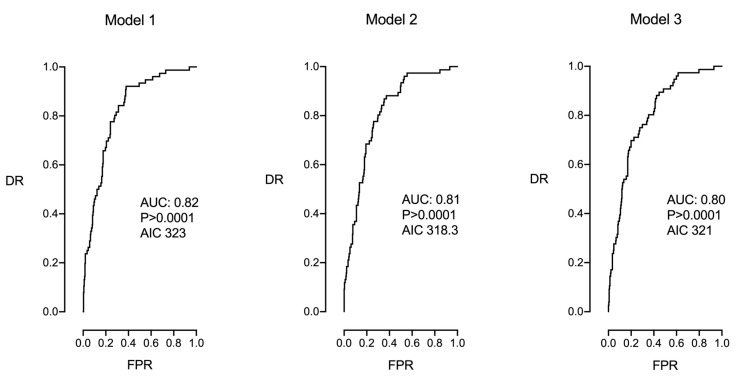
Multivariable analysis in which models 1, 2, and 3 were evaluated. Model 1 included all plausible parameters of the univariable analysis. Model 2 included only the significant parameters of model 1. Model 3 included the significant parameters of model 1, with the addition of fetal gender.

Table 3 and Figure 1 show the multivariable analysis in which three models (1–3) were evaluated. Model 1 included all plausible parameters of the univariable analysis. However, only maternal age (*p* < 0.01), parity (*p* > 0.00001), EFW centile (*p* < 0.01), cervical length (*p* < 0.05), and posterior occiput position were significant. The model obtained an AIC of 323 and an AUC of 0.82 (95% CI 0.77–0.87, *p* < 0.0001) with detection rates of 26% and 46% for false positive rates of 5% and 10%, respectively.

Model 3 included only the significant parameters of model 1, resulting in an AIC of 321 and an AUC of 0.80 (95% CI (0.75–0.85), *p* < 0.0001), with detection rates of 28% and 39% for false positive rates of 5% and 10%, respectively.

Finally, model 2 included the significant parameters of model 1 plus fetal sex (which had shown borderline significance). In this case, fetal sex became statistically significant. This model obtained an AIC of 318.3 (the lowest) and an AUC of 0.81, 95% CI (0.76–0.86) *p* < 0.0001, with detection rates of 24% and 37% for false positive rates of 5% and 10%, respectively. This model represented the most reproducible model, obtaining the lowest AIC, and one of the best at prediction, which was only surpassed by model 1 at an AUC of 0.01. Therefore, model 2 was chosen in this study as the best prediction model for CS for FP.

## 4. Discussion

### 4.1. Background

Since the publication of the first algorithm by Bishop in 1964 [10], obstetric assessment has been evolving. Some obstetric societies have published guidelines and recommendations about the usefulness of ultrasound during labor to improve the accuracy of the obstetric examination [3,4]. The most robust scientific evidence is found at the first and second stages of labor, when ultrasound can be useful for suspected delays to or arrests of labor as well as the potential need for the performance of an instrumental delivery [4]. However, just a few associations describe the use of ultrasound before induction, like the World Association of Perinatal Medicine and the Perinatal Medicine Foundation, which advocate for its usefulness throughout the whole labor process: pre-labor, during labor, and after delivery [3]. Regarding the pre-induction stage, ultrasound examination is recommended in order to understand the condition of the baby and the birth canal, as sonographic parameters could be better predictors than Bishop’s score [3,4].

### 4.2. Summary of Findings

This study suggests a simple model that includes clinical and sonographic variables for predicting CS for FP prior to labor induction, with high reproducibility and accuracy. The variables included in the model were maternal age, parity, fetal sex, EFW centile, cervical length, and posterior position of the fetal head. An AIC of 318.3 and an AUC of 0.81 were achieved.

This model may be applied to individualize the management of patients, adjusting induction protocols to the probability of a vaginal delivery.

### 4.3. The Use of Perineal Ultrasound

The novelty of our model is that it includes data from perineal ultrasound. Only a few models have previously included these variables [2,11], probably due to their retrospective design, since they are not usually measured before induction. In general, most studies agree on the superiority of ultrasound upon clinical vaginal exploration, both in terms of accuracy and tolerability for the patient [2,3,4,12,13]. However, the authors of this study advocate for the use of sonographic parameters alongside classical physical examination. The complementarity of both techniques guarantees a better obstetric evaluation.

Many sonographic parameters have been described in the literature: angle of descent, head-symphysis distance, head-perineum distance, midline angle, fetal occiput spine angle, etc. [3,4]. We disregarded head-symphysis distance and head-perineum distance due to their poor reliability [14]. In contrast, we selected the variable angle of descent for its accuracy, reproducibility, and low interobserver variability [15]. Several studies have underlined the usefulness of this parameter. In a study including nulliparous women, the authors demonstrated that if the pre-induction angle of descent was over 92°, the probability of delivery was 94.8% [14]. The angle of descent has been most frequently evaluated in the second part of the delivery [15,16]. In this period, an angle of descent over 120° has been closely related to successful spontaneous or vacuum deliveries in 90% of patients [16]. Interestingly, our multivariate analysis did not show statistical significance for the angle of descent, probably because we evaluated it too soon in the labor process instead of at the end, when it is known to exhibit better prediction ability [15,16].

This is consistent with other ultrasonographic parameters not included in this study, such as the midline angle, as they have been described to evaluate the dynamics of the second stage of labor, as well as to predict the prognosis of instrumental deliveries [3,17,18,19]. However, the patients included in our study were evaluated prior to the onset of labor.

Our model also proved that the cervical length was an essential parameter for the prediction of a successful vaginal delivery. This was consistent with the earlier data [20], which underlines the solid predictive ability of the cervical length, alone or combined with parameters like the posterior cervical angle [21].

Finally, EFW and posterior occiput position were also selected by our model. This agreed with previous studies relating the posterior occiput position and EFW with the risk of CS [6,7,22]. Moreover, most labor wards have ultrasound facilities and trained personnel to easily obtain the EFW and posterior occiput position before induction.

### 4.4. Clinical Implications

Considering the time of induction, our results indicated that the interval to 42 weeks did not influence the success of induction, suggesting that the possibility of a vaginal delivery would not be lower at 39–40 weeks than at 40–41 weeks. However, conclusions cannot be made about the interval 41–42 weeks as our hospital protocol indicates induction for pregnancy in the process of prolongation at 41 weeks at the latest, not reaching 42 weeks of pregnancy.

This would support previous research advocating for universal induction at 39 to improve the results of the perinatal outcome [23,24]. However, the decision should be agreed with the patient, since induction (especially in nulliparous women) also entails risks [23,24,25,26], such as shoulder dystocia [23]. A practical consequence of this would be that the success of induction would simply depend on the obstetric conditions. In this regard, and to evaluate them properly, some studies have advocated for the use of clinical examination, concluding that only this methodology can accurately predict vaginal delivery [11]. Our model is in line with this, but also objectifies data using pelvic ultrasound, available in all clinical settings. We acknowledge that the proposed model is pending validation. In this regard, very few published models have been externally validated [27,28]. Many authors have proposed models to predict a successful delivery. However, most were retrospective and had poor reproducibility [20,29,30,31]. In a Spanish study, external validation of 12 prediction models was performed using a cohort of 468 patients undergoing labor induction [28]. The authors concluded that the AUC of prediction ranged from 0.596 to 0.773. The model with the highest predictive power was that of Levine et al. (AUC 0.773), followed by those of Hernandez et al. (AUC 0.762) and Rossi et al. (AUC 0.752) [28]. However, another study evaluating 78 multivariate models concluded that none of them should be applied clinically [27]. Systematic reviews agree with that conclusion: there is no recommendation for use in clinical practice since the studies published so far present significant heterogeneity in design, selection criteria, sample size, included variables, and definition of the outcomes [20,29].

Some authors have raised concerns regarding the possibility of an increase in the frequency of CS when predictive models are employed. However, some studies have come to the opposite conclusion. In one study, the authors observed a 6% risk reduction in maternal morbidity and an 8% risk reduction in CS delivery achieved through the clinical use of the model devised by Levine et al. [32,33]. Moreover, when they divided the population according to the risk of FP, they observed no cases of CS in the low-risk group (<20%) [32]. Therefore, having that information empowered the patient and the professional to achieve a vaginal delivery. In addition, in the high-risk group (>60%), the rate of CS was neither reduced nor increased, and the induction time was reduced by 5 h, avoiding therapeutic overzealousness. Finally, in the intermediate-risk group (40–60%), the CS rate even decreased [32].

We consider that our model, once validated, might be applied similarly in clinical practice, thus being able to individualize protocols according to the individual risk of CS for FP.

### 4.5. Strengths and Limitations

The main strengths of our model are the prospective design and the inclusion of perineal and pelvic ultrasound data, although not all expected variables were selected. The limitations include the absence of new sonographic parameters like cervical electrography [21,34] and biochemical markers like fetal fibronectin, IGFBP-1, Activin-A, or Interleukin-6 and 8 [35,36,37]. However, the authors did not include them as the aim was to create a model that could be useful and generalizable to all types of hospitals.

## 5. Conclusions

Prediction of CS for FP prior to labor induction may be achieved using the following simple ultrasound parameters and clinical data: maternal age, parity, fetal sex, EFW centile, cervical length, and posterior position of the fetal head. This study presents a reproducible and accurate model that could be used to guide the individual management of patients, adjusting induction protocols to each patient’s likelihood of vaginal delivery.

## Figures and Tables

**Table 1:** (**a**) Description of the study population (N = 387): Clinical and demographic characteristics. (**b**) Outcomes.

(a)				
	1-All Pregnancies (N = 387)	2-Other Mode of Delivery (N = 311)	3-Cesarean for Failure to Progress (N = 76)	2 vs. 3 *
	Median (1st, 3rd quartile)	Median (1st, 3rd quartile)	Median (1st, 3rd quartile)	*p*-value
Maternal age in years	34 (30, 38)	34 (30, 38)	35 (31, 39)	<0.05
Maternal pre-pregnancy weight (kgs)	67 (59, 75)	67 (59, 76)	66.7 (58, 75.4)	NS
Maternal height (cms)	164 (160, 168)	164 (159, 168)	165 (160, 168)	NS
Maternal Body Mass Index, Kg/m^2^	24.6 (22, 27.9)	24.6 (21.8, 27.9)	24.5 (22.3, 27.6)	NS
Number of gestations	2 (1, 3)	2 (1, 3)	1 (1, 2)	<0.0001
Parity	0 (0, 1)	0 (0, 1)	0 (0, 0)	<0.0001
Gestational age at examination (weeks + days)	40 + 4 (39 + 6, 40 + 6)	40 + 4 (39 + 6, 40 + 6)	40 + 4 (39 + 6, 40+ 6)	NS
Gestational age at delivery (weeks + days)	40 + 5 (40 + 0, 41 + 0)	40 + 5 (40 + 0, 41 + 0)	40 + 5 (40 + 0, 41 + 0)	NS
Estimated fetal weight (grams)	3445 (3161, 3684)	3414 (3150, 3645)	3545 (3209, 3864)	<0.01
Estimated fetal weight centile	62(31, 85)	58 (30, 83)	70 (42.2, 94.7)	<0.01
Cervical length	27.7 (8.9); 28 (21, 33)	27.1 (9.1); 28 (20, 33)	30.5 (25, 35)	<0.01
Angle of descent	104 (96, 113)	105 (97, 115)	103.5 (89.2, 112)	<0.05
Interval to 42 weeks (days)	9 (7, 14)	9 (7, 14)	9 (7, 14)	NS
	N (%)	N (%)	N (%)	
Posterior position	165 (42.6)	116 (37.3)	49 (64.5)	<0.0001
Smoking	30 (7.7)	26 (8.4)	4 (5.2)	NS
Birth (>40 weeks)	274 (70.8)	219 (70.4)	60 (78.9)	NS
Male gender	203 (52.4)	157 (50.5)	46 (60.5)	NS
Type of induction				
Balloon (mechanical induction)	26 (6.7)	22 (7.1)	4 (5.3)	NS
Prostaglandin E2 (dinoprostone)	350 ()	279 (89.7)	71 (93.4)	NS
Oxytocin	11 (2.8)	10 (3.2)	1 (1.3)	NS
**(b)**				
	**1-All Pregnancies** **(N = 387)**	**2-Other Mode of Delivery** **(N = 311)**	**3-Cesarean for Failure to Progress (N = 76)**	2 vs. 3 *
	Median (1st, 3rd quartile)	Median (1st, 3rd quartile)	Median (1st, 3rd quartile)	*p*-value
Induction length (exam–delivery interval in hours)	24 (13 35)	22.0 (11.2); 20 (12, 32)	33.8 (7.8); 36 (29.7, 39)	<0.0001
Birth weight (grams)	3450 (3160, 3735)	3400 (3120, 3680)	3563 (3198, 3848)	<0.05
Birth weight centile	47 (19, 77)	44 (19,72)	57 (29.2, 87)	<0.05
	N (%)	N (%)	N (%)	
Apgar < 7 at 5 min	2 (0.5)	2 (0.6)	0 (0)	NS
Arterial pH < 7.10	13 (3.3)	12 (3.8)	1 (1.3)	NS
Mode of birth				
Cesarean section (failure to progress)	76 (19.6)	0 (0)	76 (100)	<0.0001
Cesarean section (intrapartum fetal compromise)	31 (8)	31 (10)	0 (0)	<0.01
Assisted vaginal delivery	82 (21.2)	82 (26.4)	0 (0)	<0.0001
Spontaneous vaginal delivery	198 (51.2)	198 (63.7)	0 (0)	<0.0001
Neonatal transferral to intensive care unit	3 (0.8)	3 (0.96)	0 (0)	NS

Notes: * Mann-Whitney U test, SD: standard deviation, NS: not significant.

**Table 2:** Univariable models for the prediction of cesarean section due to failure to progress in pregnancies undergoing induction of labor.

	b-Coefficient	SE	OR (95% CI)	OR *p*-Value	AUC	AUC *p*-Value
Maternal age	0.049	0.02	1.05 (1.00, 1.10)	<0.05	0.58	<0.05
Parity	−1.410	0.32	0.24 (0.13, 0.46)	<0.0001	0.67	<0.0001
Maternal weight	0.004	0.009	1.00 (0.99, 1.02)	NS	0.51	NS
Maternal height	0.009	0.02	1.01 (0.97, 1.05)	NS	0.51	NS
Fetal gender (male)	0.408	0.26	1.50 (0.90, 2.51)	NS	0.55	NS
EFW centile	0.012	0.004	1.01 (1.00,1.02)	<0.01	0.60	<0.01
Cervical length	0.041	0.01	1.04 (1.01, 1.07)	<0.01	0.60	<0.01
Angle of descent	−0.023	0.01	0.98 (0.96, 1.00)	<0.05	0.57	<0.05
Posterior position	1.115	0.27	3.05 (1.81, 5.15)	<0.0001	0.59	<0.01
Interval to 42 weeks	−0.009	0.02	0.99 (0.95, 1.03)	NS	0.51	NS

Notes: SE: standard error, OR: Odds ratio, NS: not significant.

**Table 3:** Multivariable models for the prediction of cesarean section due to failure to progress in pregnancies undergoing induction of labor.

	β-Coefficient	SE	OR (95% CI)	OR *p*-Value
**Model 1**. All studied parameters.				
Maternal age	0.076	0.03	1.08 (1.02, 1.14)	<0.01
Parity	−1.591	0.35	0.20 (0.10, 0.40)	<0.00001
Maternal weight	−0.0004	0.01	1.00 (0.98, 1.02)	NS
Maternal height	−0.026	0.03	0.97 (0.93, 1.03)	NS
Fetal gender (male)	0.580	0.30	1.79 (1.00, 3.20)	NS *
EFW centile	0.016	0.01	1.02 (1.01, 1.03)	<0.01
Cervical length	0.038	0.02	1.04 (1.00, 1.08)	<0.05
Angle of descent	−0.019	0.01	0.98 (0.96, 1.00)	NS
Posterior position	1.083	0.30	2.95 (1.65, 5.28)	<0.001
Interval to 42 weeks	−0.006	0.03	0.99 (0.94, 1.05)	NS
Intercept	−0.107			
AIC = 323, AUC: 0.82, 95% CI (0.77–0.87), *p* < 0.0001, DR 26% for a FPR of 5%, DR 46% for a FPR of 10%.				
**Model 2**. Significant parameters plus fetal gender (borderline significance).				
Maternal age	0.065	0.03	1.07 (1.013, 1.12)	<0.05
Parity	−1.562	0.34	0.21 (0.11, 0.41)	<0.00001
Fetal gender (male)	0.628	0.29	1.87 (1.05, 3.33)	<0.05
EFW centile	0.015	0.01	1.02 (1.00, 1.03)	<0.01
Cervical length	0.046	0.02	1.05 (1.01, 1.08)	<0.01
Posterior position	1.104	0.29	3.02 (1.70, 5.36)	<0.001
Intercept	−6.270			
AIC = 318.3, AUC: 0.81, 95% CI (0.76–0.86), *p* < 0.0001, DR 24% for a FPR of 5%, DR 37% for a FPR of 10%.				
**Model 3**. Significant parameters.				
Maternal age	0.062	0.03	1.06 (1.01, 1.12)	<0.05
Parity	−1.504	0.33	0.22 (0.12, 0.43)	<0.00001
EFW centile	0.015	0.01	1.02 (1.00, 1.03)	<0.01
Cervical length	0.043	0.02	1.04 (1.01, 1.08)	<0.01
Posterior position	1.080	0.29	2.94 (1.66, 5.21)	<0.001
Intercept	−5.747			
AIC = 321, AUC: 0.80, 95% CI (0.75–0.85), *p* < 0.0001, DR 28% for a FPR of 5%, DR 39% for a FPR of 10%.				

Notes: * Borderline significance 0.05079, SE: standard error, OR: Odds ratio, NS: not significant.

## Data Availability

Data will be obtained by reaching out to the authors while ensuring the privacy of the patients is maintained.

## Abbreviations

CS: cesarean section; FP: failure to progress; EFW: estimated fetal weight; AUC: area under the curve; AIC: Akaike Information Criterion; CI: confidence interval.

## References

[1] Carlson N., Ellis J., Page K., Amore A.D., Phillippi J. (2021). Review of Evidence-Based Methods for Successful Labor Induction. J. Midwifery Women’s Health.

[2] Alvarez-Colomo C., Gobernado-Tejedor J.A. (2015). The validity of ultrasonography in predicting the outcomes of labour induction. Arch. Gynecol. Obstet..

[3] Rizzo G., Ghi T., Henrich W., Tutschek B., Kamel R., Lees C.C., Mappa I., Kovalenko M., Lau W., Eggebo T. (2022). Ultrasound in labor: Clinical practice guideline and recommendation by the WAPM-World Association of Perinatal Medicine and the PMF-Perinatal Medicine Foundation. J. Perinat. Med..

[4] Ghi T., Eggebø T., Lees C., Kalache K., Rozenberg P., Youssef A., Salomon L.J., Tutschek B. (2018). ISUOG Practice Guidelines: Intrapartum ultrasound. Ultrasound Obstet. Gynecol..

[5] Fuster-Rojas S.I., Valero-Domínguez J. (2014). Inducción del parto sin cesárea previa. Obstetricia y Ginecología, guía de actuación.

[6] Akmal S., Kametas N., Tsoi E., Howard R., Nicolaides K.H. (2004). Ultrasonographic occiput position in early labour in the prediction of caesarean section. BJOG Int. J. Obstet. Gynaecol..

[7] Kamel R.A., Negm S.M., Youssef A., Bianchini L., Brunelli E., Pilu G., Soliman M., Nicolaides K.H. (2021). Predicting cesarean delivery for failure to progress as an outcome of labor induction in term singleton pregnancy. Am. J. Obstet. Gynecol..

[8] Milner J., Arezina J. (2018). The accuracy of ultrasound estimation of fetal weight in comparison to birth weight: A systematic review. Ultrasound.

[9] Coutinho C.M., Sotiriadis A., Odibo A., Khalil A., D’Antonio F., Feltovich H., Salomon L.J., Sheehan P., Napolitano R., Berghella V. (2022). ISUOG Practice Guidelines: Role of ultrasound in the prediction of spontaneous preterm birth. Ultrasound Obstet. Gynecol..

[10] Bishop E.H. (1964). Pelvic Scoring for Elective Induction. Obstet Gynecol..

[11] Reis F., Gervasi M., Florio P., Bracalente G., Fadalti M., Severi F., Petraglia F. (2003). Prediction of successful induction of labor at term: Role of clinical history, digital examination, ultrasound assessment of the cervix, and fetal fibronectin assay. Am. J. Obstet. Gynecol..

[12] Rane S.M., Guirgis R.R., Higgins B., Nicolaides K.H. (2004). The value of ultrasound in the prediction of successful induction of labor. Ultrasound Obstet. Gynecol..

[13] Usman S., Barton H., Wilhelm-Benartzi C., Lees C.C. (2018). Ultrasound is better tolerated than vaginal examination in and before labour. Aust. N. Z. J. Obstet. Gynaecol..

[14] Gillor M., Vaisbuch E., Zaks S., Barak O., Hagay Z., Levy R. (2016). Transperineal sonographic assessment of angle of progression as a predictor of successful vaginal delivery following induction of labor. Ultrasound Obstet. Gynecol..

[15] Barbera A.F., Pombar X., Perugino G., Lezotte D.C., Hobbins J.C. (2009). A new method to assess fetal head descent in labor with transperineal ultrasound. Ultrasound Obstet. Gynecol..

[16] Kalache K.D., Dückelmann A.M., Michaelis S.M., Lange J., Cichon G., Dudenhausen J.W. (2009). Transperineal ultrasound imaging in prolonged second stage of labor with occipitoanterior presenting fetuses: How well does the ‘angle of progression’ predict the mode of delivery?. Ultrasound Obstet. Gynecol..

[17] Hinkson L., Henrich W., Tutschek B. (2020). Intrapartum ultrasound during rotational forceps delivery: A novel tool for safety, quality control, and teaching. Am. J. Obstet. Gynecol..

[18] Henrich W., Dudenhausen J., Fuchs I., Kämena A., Tutschek B. (2006). Intrapartum translabial ultrasound (ITU): Sonographic landmarks and correlation with successful vacuum extraction. Ultrasound Obstet. Gynecol..

[19] Tutschek B., Braun T., Chantraine F., Henrich W. (2010). A study of progress of labour using intrapartum translabial ultrasound, assessing head station, direction, and angle of descent. BJOG Int. J. Obstet. Gynaecol..

[20] D’Souza R., Ashraf R., Foroutan F. (2021). Prediction models for determining the success of labour induction: A systematic review and critical analysis. Best Pract. Res. Clin. Obstet. Gynaecol..

[21] Costas T., de la O Rodríguez M., Sánchez-Barba M., Alcázar J.L. (2023). Predictive Value of Cervical Shear Wave Elastography in the Induction of Labor in Late-Term Pregnancy Nulliparous Women: Preliminary Results. Diagnostics.

[22] Prado C.A.d.C., Júnior E.A., Duarte G., Quintana S.M., Tonni G., Cavalli R.d.C., Marcolin A.C. (2016). Predicting success of labor induction in singleton term pregnancies by combining maternal and ultrasound variables. J. Matern.-Fetal Neonatal Med..

[23] Hong J., Atkinson J., Mitchell A.R., Tong S., Walker S.P., Middleton A., Lindquist A., Hastie R. (2023). Comparison of Maternal Labor-Related Complications and Neonatal Outcomes Following Elective Induction of Labor at 39 Weeks of Gestation vs Expectant Management: A Systematic Review and Meta-analysis. JAMA Netw. Open.

[24] Burrows A., Finkenzeller K., Pudwell J., Smith G. (2022). Elective Induction of Labour at 39 Weeks Compared with Expectant Management in Nulliparous Persons Delivering in a Community Hospital. J. Obstet. Gynaecol. Can..

[25] Sinkey R.G., Lacevic J., Reljic T., Hozo I., Gibson K.S., Odibo A.O., Djulbegovic B., Lockwood C.J. (2018). Elective induction of labor at 39 weeks among nulliparous women: The impact on maternal and neonatal risk. PLoS ONE.

[26] Marrs C., La Rosa M., Caughey A., Saade G. (2019). Elective Induction at 39 Weeks of Gestation and the Implications of a Large, Multicenter, Randomized Controlled Trial. Obstet. Gynecol..

[27] Collins G.S., A de Groot J., Dutton S., Omar O., Shanyinde M., Tajar A., Voysey M., Wharton R., Yu L.-M., Moons K.G. (2014). External validation of multivariable prediction models: A systematic review of methodological conduct and reporting. BMC Med. Res. Methodol..

[28] López-Jiménez N., García-Sánchez F., Hernández-Pailos R., Rodrigo-Álvaro V., Pascual-Pedreño A., Moreno-Cid M., Delgado-Rodríguez M., Hernández-Martínez A. (2021). Risk of caesarean delivery in labour induction: A systematic review and external validation of predictive models. BJOG Int. J. Obstet. Gynaecol..

[29] Meier K., Parrish J., D’Souza R. (2019). Prediction models for determining the success of labor induction: A systematic review. Acta Obstet. Gynecol. Scand..

[30] Gonen R., Degani S., Ron A. (1998). Prediction of successful induction of labor: Comparison of transvaginal ultrasonography and the Bishop score. Eur. J. Ultrasound.

[31] Kolkman D.G.E., Brinkhorst S.J., van der Post J.A.M., Pajkrt E., Opmeer B.C., Mol B.W.J., Verhoeven C.J.M. (2013). The Bishop Score as a Predictor of Labor Induction Success: A Systematic Review. Am. J. Perinatol..

[32] Hamm R.F., McCoy J., Oladuja A., Bogner H.R., Elovitz M.A., Morales K.H., Srinivas S.K., Levine L.D. (2020). Maternal Morbidity and Birth Satisfaction After Implementation of a Validated Calculator to Predict Cesarean Delivery During Labor Induction. JAMA Netw. Open.

[33] Levine L.D., Downes K.L., Parry S., Elovitz M.A., Sammel M.D., Srinivas S.K. (2017). A validated calculator to estimate risk of cesarean after an induction of labor with an unfavorable cervix. Am. J. Obstet. Gynecol..

[34] Lu J., Cheng Y.K.Y., Ho S.Y.S., Sahota D.S., Hui L.L., Poon L.C., Leung T.Y. (2019). The predictive value of cervical shear wave elastography in the outcome of labor induction. Acta Obstet. Gynecol. Scand..

[35] Lau S.L., Kwan A., Tse W.T., Poon L.C. (2021). The use of ultrasound, fibronectin and other parameters to predict the success of labour induction. Best Pract. Res. Clin. Obstet. Gynaecol..

[36] Garite T.J., Casal D., Garcia-Alonso A., Kreaden U., Jimenez G., Ayala J.A., Reimbold T. (1996). Fetal fibronectin: A new tool for the prediction of successful induction of labor. Am. J. Obstet. Gynecol..

[37] Blanch G., Oláh K.S., Walkinshaw S. (1996). The presence of fetal fibronectin in the cervicovaginal secretions of women at term—Its role in the assessment of women before labor induction and in the investigation of the physiologic mechanisms of labor. Am. J. Obstet. Gynecol..

